# Perceived social cohesion and depressive symptoms among internal migrants in China: The mediating role of social adaptation

**DOI:** 10.3389/fpubh.2023.1096318

**Published:** 2023-02-07

**Authors:** Xiaomin Qu, Xiang Qi, Bei Wu, Jiaojiao Yu, Haidong Zhang

**Affiliations:** ^1^School of Social Development, East China University of Political Science and Law, Shanghai, China; ^2^Rory Meyer College of Nursing, New York University, New York, NY, United States; ^3^School of Customs and Public Administration, Shanghai Customs College, Shanghai, China; ^4^School of Sociology and Political Science, Shanghai University, Shanghai, China

**Keywords:** social cohesion, social adaptation, depression, migration, mental health disparities

## Abstract

**Background:**

Internal migrants are exposed to higher risks of depressive symptoms due to migration-related stress. It has been recognized that perceived neighborhood social cohesion has direct and indirect associations with depressive symptoms. However, the pathway from perceived social cohesion to internal migrants' depressive symptoms was less discussed.

**Objectives:**

To assess mental health disparities among internal migrants. To investigate the association between social cohesion and depressive symptoms among urban-to-urban and rural-to-urban migrants and to examine the mediating role of social adaptation.

**Methods:**

Data from the “2017 Urbanization and New Migrant Survey” was used, including 2,584 internal migrants age 18–65 from 10 cities in China. Social cohesion was measured by a six-item modified Community-level Cohesion Scale. Depressive symptoms was measured using the Center for Epidemiological Studies Depression Scale, and social adaptation was assessed by a single-item question of migrants' adaptation to local life. Multivariate linear regression models were used to examine the association between social cohesion and depressive symptoms. Baron and Kenny's mediation tests were conducted to examine the mediating role of social adaptation on the association. All analyses were adjusted using sampling weights to account for this survey's sampling design.

**Results:**

Rural-to-urban migrants were found to have more clinically significant depressive symptoms, lower perceived social cohesion, and fair or low social adaptation than urban-to-urban migrants (all *p* < 0.001). Being rural-to-urban migrants as compared with urban-to-urban migrants [Odds Ratio (OR) = 1.46, 95% Confidence Interval (CI) = 1.456, 1.461, *p* < 0.001], had lower perceived social cohesion (OR = 1.46, 95% CI = 1.458, 1.463, *p* < 0.001), and poorer social adaptation (OR = 1.94, 95% CI = 1.932, 1.941, *p* < 0.001), are associated with higher odds of having clinically significant depressive symptoms. Social adaptation partially mediated the association between social cohesion and depressive symptoms by explaining 15.39% of its effect for urban-to-urban migrants and 18.97% for rural-to-urban migrants.

**Conclusions:**

Findings from this study reveal mental health inequalities among internal migrants and demonstrate the importance of social adaption on the association between social cohesion and depressive symptoms. Social strategies and public policies are needed to build a more cohesive community that serves both local residents and internal migrants, especially rural-to-urban migrants.

## Introduction

Depressive symptoms are common mental health problems among migrants in many countries. This issue could be exacerbated due to an increase in the migrant population around the world (1). Internal migrants are individuals who migrate between regions within one country (2). Due to China's economic development since 1978, internal migrants have increased dramatically. Internal migrants reached 244 million in 2017, accounting for more than 17.5% of the total population (3). For migrants, depressive symptoms are especially associated with their intention to settle in the host place (4). However, the mental health status of internal migrants did not receive much attention (5, 6).

Increasing evidence suggests that migrants experience higher levels of depressive symptoms than native-born residents (7–10) because of migration-related changes, such as separation from family, reducing size in social support, and weakened social ties (11). In China, migration has been regarded as a stressful process (10, 12). For migrants, due to the housing registration system (i.e., *Hukou*) that limits their access to employment, education, housing, and health insurance (13, 14), they are more likely to be exposed to social stress and social exclusion (6, 11, 12, 15) that may lead to a higher level of depressive symptoms. There are two major internal migration patterns in China: one is from rural-to-urban migration and the other from urban-to-urban migration (16). However, most previously studies that examined mental health status were only focused on rural-to-urban migrants (5, 11, 17, 18).

In addition, it has long been recognized that individual and family level social factors, such as family socioeconomic status, social capital, and social support, have direct relationships with depressive symptoms (19–24), while little attention has been paid on the role of social cohesion, as a community-level social factor that is associated with depressive symptoms among internal migrants. Social cohesion indicates the inclusion and integration of a community (25, 26). As an important environmental factor, social cohesion is related to individuals' psychological wellbeing (27, 28). In a migrant community, social cohesion has an impact on internal migrants' positive interactions including obtaining social support and help, or negative interactions such as suffering from social stress and/or exclusion from native-born residents. However, little attention has been paid to the association of social cohesion, as a community-level social factor, with depressive symptoms among internal migrants in China.

The rapid increase of the migration population, intertwined with dramatic social transition, suggests that Chinese society has reached a critical stage in mental health care challenges. Nevertheless, little is known about how social environmental context, beyond intra-personal factors, affects depressive symptoms among internal migrants in China. To address these knowledge gaps, this study aims to investigate the association between social cohesion and depressive symptoms among urban-to-urban and rural-to-urban migrants and to examine the mediating role of social adaptation, using the data drawn from the “2017 Urbanization and New Migrant Survey,” a large-scale sample with 2,584 internal migrants from 10 cities in China. Gaining a better understanding of the pathways would provide a better knowledge on community-level social factors that are associated with depressive symptoms and help develop social strategies and public policies in addressing mental health issues among migrant population.

## Literature review

### Social inequalities: Disparities in depressive symptoms within migrants

When studying the migrant-related health disparities, scholars often use a native-born population in the host society as the reference group. Most of the studies demonstrated that migrants had poorer mental health, e.g., depressive symptoms, than native-born residents (6, 11, 29, 30). Depressive symptoms may lead to a series of complications that affect individual's quality of life and cause other critical health issues such as suicide, frailty, functional disability, and mortality (31–33). For migrants (including both international and internal migrants), depressive symptoms are also associated with their intention to settle down in the host place (4).

However, most studies on health outcomes in China only focused on the rural-to-urban migrants (6, 11, 17, 18). These studies suggest that there are significant mental health disparities within migrants. Furthermore, more information about migration status, e.g., the housing registration status of migrants, and reasons for migration, should be considered when examining the mental health disparities among migrants. In China, the most important characteristic that distinguishes the patterns of migration from those in other countries is the housing registration system (*Hukou* system) (13, 34). Since *Hukou* is linked with entitlement and benefits for individuals, whether migrants come from urban or rural is an indicator for their education background, financial status, and social support (13, 34–36), which may result in different mental health outcomes, such as depressive symptoms. To assess disparities in depressive symptoms among migrants, we proposed the following hypothesis:

Hypothesis 1: Rural-to-urban migrants are more likely to have a higher level of depressive symptoms than urban-to-urban migrants.

### Social cohesion in reducing depressive symptoms

There is no doubt that environmental context plays a crucial role in shaping psychological wellbeing (27, 37, 38). When investigating risk and protective factors for depressive symptoms of migrants, it is likely that community-level factors are at play.

It is well-recognized that individual and neighborhood-level factors, such as neighborhood socioeconomic status, social capital, and social support, have strong associations with residents' depressive symptoms (19–22, 37–43). A related concept of particular importance to the migrant community is social cohesion (27, 44). Social cohesion refers to communal bonds characterized by altruism, reciprocity, and shared norms and values (27). It generates mutual trust and support, collective efficacy, and a sense of belonging, which are all conducive to improved mental health (27, 45). Previous studies show that social cohesion was defined as a neighborhood-level factor, and lower neighborhood social cohesion was linked to a variably of health outcomes, such as depression and mortality (46–52).

Neighborhood social cohesion is the network of relationships, shared values, and norms of residents in a neighborhood and it shares some similarities to an individual's social network (53). Social cohesion, however, exists in a larger field. It accounts for value systems, degree of social interaction, and considers the cohesion of a broader community rather than cohesion within a neighborhood or a small group of individuals. However, little attention has been paid to the association of social cohesion, as a community-level social factor, with depressive symptoms among internal migrants in China. In a migrant community, social cohesion refers to the quality of social interactions which is closely related to individuals' mental health status (11, 20, 22, 41, 54). In a more cohesive community, migrants may feel a stronger sense of inclusion. Social cohesion has an impact on internal migrants' positive interactions including obtaining social support and help, or negative interactions such as suffering from social stress and/or exclusion from native-born residents. In a community where native-born residents and migrants trust each other, they will have more social connections and interactions. For migrants, the trust they have built will make it easier for them to establish new social ties and social networks that are important to adapt to a new life and maintain a good psychological status (20, 21, 42). In addition, a higher level of social cohesion would lead to less social stress or social exclusion, which may result in fewer depressive symptoms (6, 11, 12). Furthermore, in a well-integrated community, native-born residents and migrants are more likely to provide mutual social support, which is a protective factor against depressive symptoms (22, 41). Even if some migrants suffer from depressive symptoms, they are more likely to be able to receive timely assistance in a more cohesive community. To investigate the associations between social cohesion and depressive symptoms of both urban-to-urban and rural-to-urban migrants, here is the second hypothesis:

Hypothesis 2: A lower level of perceived social cohesion is associated with more depressive symptoms among both urban-to-urban and rural-to-urban migrants.

### Social adaptation: Pathways from social cohesion to depressive symptoms

Although recent studies have shown the correlation between social cohesion and health outcomes (49, 50, 52, 55, 56), limited studies have been conducted to examine the mediating role of social adaptation on the association between social cohesion and migrants' mental health. Increasing attention has been paid to improve social adaptation as an effort to improve individual's mental health (57, 58). Social adaptation concerns the interactions between an individual and the environment. It refers to the performance in the activities of daily intercultural living (59) and involves the intercultural competence with emphasis on behavioral domains. As an important indicator of migrants' integration into the host community, social adaptation would mediate the pathways from social cohesion to depressive symptoms. Individual's allostatic load would be lower in a highly cohesive community where internal migrants have more positive interactions with local residents and a higher sense of being trusted and belonging, Individuals are more likely to “doing well” in the activities of daily intercultural living in the host city (59). Additionally, “doing well” in social adaptation may help buffer the effects of social stress and social exclusion they may face in the migration process (6, 11, 12); thus, avoiding the occurrence of psychological disorders. Therefore, no such studies have been conducted to examine whether social adaptation is a factor that could mediate between social cohesion and depressive symptoms. Thus, we propose the following hypothesis:

Hypothesis 3: Social adaptation mediates the association between social cohesion and depressive symptoms for both urban-to-urban and rural-to-urban migrants.

## Methods

### Data source and study sample

Data were drawn from the “2017 Urbanization and New Migrant Survey,” a cross-sectional survey focusing on policy issues such as population migration, social mobility, and social integration in Chinese adults. A multi-stage stratified sampling strategy was used, and data were collected from 10 cities in China, including economically developed cities (GDP per capita of is more than 100,000 RMB), such as Zhengzhou, Tianjin, Xiamen, Guangzhou, and Changsha, and less developed ones (GDP per capita of is <100,000 RMB), including Harbin, Changchun, Yanji, Shenyang, and Anshan. Data were collected during in-home interviews by well-trained interviewers. Inclusion criteria for participation were: (1) full-time residence in this city >6 months in the past year; (2) aged 18–65 years; and (3) capable of communicating answers to interview questions and giving consent.

A sample of 2,752 adult internal migrants (i.e., the movement of people between usual residences within national states) were drawn from the survey data. After excluding 168 adults with missing values, the final analytical sample consisted of 2,584 internal migrants. Among them, 1,152 reported living in another urban area before moving to their current locations, defined as urban-to-urban migrants, and 1,432 reported living in a rural area before moving to their current locations, defined as rural-to-urban migrants.

### Measures

#### Dependent variables: Depressive symptoms

Depressive symptoms were measured using the original 20-item version of the Center for Epidemiological Studies Depression Scale (CES-D), a widely used screening measure for depressive symptoms (60). The CES-D assesses how often a person has experienced symptoms of depression, such as restless sleep, poor appetite, and feeling lonely over the past week. Each scale item is scored from 0 to 3, with a higher score representing greater depressive symptom severity. The potential range of the scale is 0–60. A cut-off score of 16 or higher is generally used to determine clinically significant depressive symptoms (61). The Cronbach's alpha for the total scale was 0.873, indicating favorable internal-consistency.

#### Independent variables: Social cohesion

The concept of perceived social cohesion in this study focuses on native-born residents' intergroup relationships with internal migrants. It refers to whether internal migrants could perceive a high or low sense of being welcomed and trusted by local residents or belonging to the community (62). With reference to the neighborhood-level cohesion scale (63–65), we generated a summary variable based on six questions, including “How much do you think native-born people are willing to (1) work with you; (2) talk with you; (3) be your neighbors; (4) make friends with you; (5) be your relatives; (6) manage the community together with you?” Each item was scored 1–5, 1 = very unwilling to, 2 = unwilling to, 3 = fair, 4 = willing to, and 5 = very willing to, with lower values representing lower perceived social cohesion. The potential range of the scale is 0–30. The six items showed good internal consistency in this study (Cronbach's α = 0.929). Considering that the median score on the social cohesion scale was 24, we then dichotomized the variable, with scores below 24 indicating a low perception of social cohesion.

#### Mediating variables: Social adaptation

Social adaptation was assessed based on the single-item question: “How much do you think you have adapted to local life?” The five-point response to this question—very poor, poor, fair, good, and very good—was then dichotomized into participants who reported fair or poor social adaptation vs. all else. Approximately 13% of the sample was categorized into the fair or poor category. We chose the fair or poor cut-off point because this is a qualitatively different group of migrants than those who are “doing well” and reporting good, or very good social adaptation (59).

#### Control variables

We included a set of confounding variables associated with depressive symptoms (7–10). Specifically, we controlled for: (i) socio-demographic characteristics, including gender (1 = female, 0 = male), age (in years), marital status (1 = married or with a partner, 0 = single or without a partner), education (years of schooling), and income (log-transformed); (ii) health behaviors, including smoking (1 = current smoker, 0 = non-smoker), alcohol consumption (1 = less than once a month, 2 = one to three times a month, 3 = one or two times a week, 4 = three to four times a week, and 5 = almost every day), and physical activity (1 = never, 2 = once a month, 3 = two or three times a month, 4 = two or three times a week, and 5 = almost every day); (iii) health status measured by self-reported physician-diagnosed chronic diseases (yes or no), including type 2 diabetes, hypertension, and heart disease. Additionally, we controlled for migration characteristics, including reasons for migration, migration time (in years), and city of residence. Reasons for migration were dichotomized into voluntary migration, which includes training or career opportunities (i.e., labor migration, occupation mobility, training and learning, and business investment), and involuntary migration for marriage or family reunion. Time since migration (in years) indicated how long migrants lived in the hosting city. The city of residence was dichotomized into more developed or less developed, depending on whether the GDP per capita of the hosting city is more than 100,000 RMB (equivalent to 14,286 US dollars).

#### Analytic strategy

We used Stata 15.0 for all statistical analysis (66). We used descriptive statistics to characterize the analytical sample separately for urban-to-urban and rural-to-urban migrants. To assess the disparities between groups, we conducted ANOVA tests for continuous variables and Chi-squared tests for categorical variables.

The bivariate logit models were used to examine whether there were significant differences in depressive symptoms between different groups. The multivariate linear regression models were used to examine the associations between social cohesion and depressive symptoms for both groups. Testing for mediation presumes a causal chain of events. The analyses cannot actually prove this causality, but they can show whether the data are in alignment with the proposed chain of events. In our analyses, we followed the procedure for establishing mediation proposed by Baron and Kenny (67). Adjusting for all covariates, we examined the mediating role of social adaptation on the association between social cohesion and depressive symptoms (67). Sobel and Goodman's methods were adapted to test the indirect effects of the mediating variables (68, 69).

#### Weighting

A multi-stage stratified sampling strategy was used, and the sampling process involved four stages: city, neighborhood, household, and individual. Ten cities were selected from seven provinces in China. At the city level, 20 neighborhoods were randomly selected from each city. At the neighborhood level, 25 households were selected from a housing registration database obtained from each neighborhood community. One individual was selected from each selected household. In this survey, the quota for migrants and non-migrants was designed to be close to 1:1. The “2017 Urbanization and New Migrant Survey” has individual-level sampling weights that take the complex sampling design into consideration. We defined the city as SU (*n* = 10), and the neighborhood as strata (*n* = 20). All analyses presented in Tables 1–**4** were adjusted for sample weights (70).

**Table 1:** Descriptive statistics by migration status (weighted).

	**Total**	**Migration status**		***p*-value**
		**Urban-to-urban**	**Rural-to-urban**	
	***N*** = **2,584**	***N*** = **1,227**	***N*** = **1,357**	
	**%/mean (SD)**	**%/mean (SD)**	**%/mean (SD)**	
**Depressive symptoms (CES-D score)**				<0.001
No (scored 1–15)	70.82	74.88	67.14	
Yes (scored 16–60)	29.18	25.12	32.86	
**Social cohesion**				<0.001
Moderate-high	61.79	68.04	56.13	
Low	38.21	31.96	43.87	
**Social adaptation**				<0.001
Good	87.57	92.01	83.54	
Fair or poor	12.43	7.99	16.46	
**Gender**				<0.001
Male	45.20	42.68	47.48	
Female	54.80	57.32	52.52	
**Age (in years)**	41.58 (13.35)	43.48 (13.85)	39.86 (12.64)	<0.001
**Marital status**				<0.001
Single or without a partner	23.61	26.12	21.33	
Married or with a partner	76.39	73.88	78.67	
**Education (years of schooling)**				<0.001
0 (illiterate)	1.63	0.38	2.76	
6 (elementary school)	6.53	1.57	11.02	
9 (middle school)	21.62	14.93	27.68	
12 (high/vocational school)	27.61	29.06	26.30	
15 (3 years college)	17.05	20.79	13.67	
16 (a bachelor' degree)	19.14	25.34	13.54	
19 (a master's degree or more)	6.41	7.94	5.03	
**Income (log-transformed)**	11.48 (1.86)	11.43 (1.80)	11.52 (1.91)	<0.001
**Smoking**				<0.001
Non-smoker	78.34	78.79	77.93	
Current smoker	21.66	21.21	22.07	
**Alcohol consumption**				<0.001
Less than once a month	76.22	77.64	74.93	
One to three times a month	8.64	8.56	8.72	
One to two times a week	7.34	6.28	8.30	
Three to four times a week	3.90	4.13	3.70	
Almost every day	3.90	3.38	4.36	
**Physical activity**				<0.001
Never	25.92	24.51	27.20	
Once a month	8.15	6.58	9.57	
Two or three times a month	18.15	19.53	16.89	
Two or three times a week	22.77	23.11	22.46	
Almost every day	25.02	26.28	23.88	
**Diabetes**				<0.001
No	96.85	95.73	97.87	
Yes	3.15	4.27	2.13	
**Hypertension**				<0.001
No	90.70	88.23	92.93	
Yes	9.30	11.77	7.07	
**Heart diseases**				<0.001
No	96.40	95.28	97.41	
Yes	3.60	4.72	2.59	
**Reasons for migration**				<0.001
Voluntary migration	62.58	50.03	73.94	
Involuntary migration	37.42	49.97	26.06	
**Time since migration (in years)**	14.03 (12.24)	15.99 (13.60)	12.26 (10.56)	<0.001
**City of residence**				<0.001
Less developed	30.78	31.14	30.45	
More developed	69.22	68.86	69.55	

Source. 2017 Urbanization and New Migrant Survey.

Comparisons between groups were done using Chi-squared tests for categorical variables and ANOVA tests for continuous variables.

CES-D, Center for Epidemiological Studies Depression Scale; SD, Standard Deviation.

## Results

### Descriptive statistics

Descriptive characteristics of the final weighted analytical sample are presented by migration status in Table 1. Rural-to-urban migrants were found to have more clinically significant depressive symptoms than urban-to-urban migrants (32.86 vs. 25.12%, *p* < 0.001). Approximately 43.87% of rural-to-urban migrants reported low social cohesion compared to 31.96% of urban-to-urban migrants. Approximately 16.46% of rural-to-urban migrants reported fair or low social adaptation compared to 7.99% of urban-to-urban migrants. Moreover, rural-to-urban migrants were less educated than urban-to-urban migrants. They tended to stay in the hosting city for a shorter duration (12.26 vs. 15.99 years, *p* < 0.001).

### Prevalence of depressive symptoms by migration status, social cohesion, and social adaptation

Weighted prevalence of depressive symptoms by migration status, social cohesion, social adaptation and covariates are presented in Table 2. Results showed that being rural-to-urban migrants as compared with urban-to-urban migrants [Odds Ratio (OR) = 1.46, 95% Confidence Interval (CI) = 1.456, 1.461, *p* < 0.001], had lower perceived social cohesion (OR = 1.46, 95% CI = 1.458, 1.463, *p* < 0.001), and poorer social adaptation (OR = 1.94, 95% CI = 1.932, 1.941, *p* < 0.001), are associated with higher odds of having clinically significant depressive symptoms. Moreover, being married or with a partner (OR = 0.68, 95% CI = 0.679,0.682, *p* < 0.001) are associated with lower odds of having clinically significant depressive symptoms. Self-reported physician-diagnosed type 2 diabetes (OR = 1.71, 95% CI = 1.707,1.723, *p* < 0.001) and heart diseases (OR = 1.18, 95% CI = 1.172,1.183, *p* < 0.001) are associated with higher odds of having clinically significant depressive symptoms.

**Table 2:** Presence of depressive symptoms by migration status, social cohesion, social adaptation, and covariates (weighted).

	***N***	**Presence of depressive symptoms (%)**	**OR**	**95% CI**	***p*-value**
**Total**	2,584	29.18			
**Migration status**					
Urban-to-urban migrants	1,227	25.12	Ref		
Rural-to-urban migrants	1,357	32.86	1.46	1.456–1.461	<0.001
**Social cohesion**					
Moderate-high	1,597	26.15	Ref		
Low	987	34.09	1.46	1.458–1.463	<0.001
**Social adaptation**					
Good	2,263	27.34	Ref		
Fair or poor	321	42.16	1.94	1.932–1.941	<0.001
**Gender**					
Male	1,168	28.34	Ref		
Female	1,416	29.89	1.08	1.076–1.080	<0.001
**Age (in years)**					
18–65	2,584	-	0.98	0.984–0.984	<0.001
**Marital status**					
Single or without a partner	610	35.49	Ref		
Married or with a partner	1,974	27.24	0.68	0.679–0.682	<0.001
**Education (years of schooling)**					
0 (illiterate)	42	27.76	Ref		
6 (elementary school)	168	30.87	1.16	1.154–1.171	<0.001
9 (middle school)	559	32.64	1.26	1.252–1.270	<0.001
12 (high/vocational school)	713	27.37	0.98	0.974–0.988	<0.001
15 (3 years college)	441	26.38	0.93	0.926–0.939	<0.001
16 (a bachelor' degree)	495	30.70	1.15	1.145–1.161	<0.001
19 (a master's degree or more)	166	26.90	0.96	0.951–0.965	<0.001
**Income (log-transformed)**					
0–16.11	2,584	-	1.03	1.033–1.034	<0.001
**Smoking**					
Nonsmoker	2,024	30.44	Ref		
Current smoker	560	24.64	0.75	0.745–0.749	<0.001
**Alcohol consumption**					
Less than once a month	1,969	29.47	Ref		
One to three times a month	223	30.06	1.03	1.026–1.032	<0.001
One to two times a week	190	25.66	0.83	0.823–0.829	<0.001
Three to four times a week	101	31.10	1.08	1.076–1.085	<0.001
Almost every day	101	26.47	0.86	0.858–0.866	<0.001
**Physical activity**					
Never	670	32.43	Ref		
Once a month	211	35.21	1.13	1.129–1.136	<0.001
Two or three times a month	469	32.21	0.99	0.987–0.992	<0.001
Two or three times a week	588	28.84	0.84	0.842–0.847	<0.001
Almost every day	646	21.98	0.59	0.585–0.588	<0.001
**Diabetes**					
No	2,503	28.80	Ref		
Yes	81	40.96	1.71	1.707–1.723	<0.001
**Hypertension**					
No	2,344	29.39	Ref		
Yes	240	27.19	0.90	0.895–0.900	<0.001
**Heart diseases**					
No	2,491	29.06	Ref		
Yes	93	32.54	1.18	1.172–1.183	<0.001
**Reasons for migration**					
Voluntary migration	1,617	29.50	Ref		
Involuntary migration	967	28.66	0.96	0.958–0.962	<0.001
**Time since migration (in years)**					
0.5–63.5	2,584	-	0.99	0.986–0.986	<0.001
**City of residence**					
Less developed	795	30.00	Ref		
More developed	1,789	28.82	0.94	0.942–0.946	<0.001

Source. 2017 Urbanization and New Migrant Survey.

Comparisons between groups were done using bivariate logit regression.

OR, Odds Ratio; CI, Confidence Interval.

### Associations between social cohesion and social adaptation

As shown in Table 3, fair or poor social adaptation was associated with a higher odd of migrants with a low perceived social cohesion for both urban-to-urban migrants (OR = 2.91, 95% CI = 2.89, 2.92, *p* < 0.001) and rural-to-urban migrants (OR = 4.12, 95% CI = 4.11, 4.13, *p* < 0.001). After adjustment for all covariates, the odds ratio reduced to 2.69 (95% CI = 2.67, 2.70, *p* < 0.001) for urban-to-urban migrants and 3.66 (95% CI = 3.65, 3.67, *p* < 0.001) for rural-to-urban migrants.

**Table 3:** Associations between social cohesion and social adaptation (weighted).

	**Urban-to-urban migrants**		**Rural-to-urban migrants**	
	**(*****N*** = **1,227)**		**(*****N*** = **1,357)**	
	**OR**	**95% CI**	**OR**	**95% CI**
Low social cohesion (unadjusted)	2.91^***^	2.89–2.92	4.12^***^	4.11–4.13
Low social cohesion (adjusted)	2.69^***^	2.67–2.70	3.66^***^	3.65–3.67

Source. 2017 Urbanization and New Migrant Survey.

Adjusted model controlled for socio-demographic characteristics, health behaviors, health status, and migration characteristics, including gender, age, marriage status, education, income, smoking, alcohol consumption, physical activity, diabetes, hypertension, heart disease, reasons for migration, migration time, and city of residence.

OR, Odds Ratio; CI, Confidence Interval.

^*^p < 0.05,

^**^p < 0.01,

*** p < 0.001.

### Associations between social cohesion and depressive symptoms

Table 4 shows the associations between social cohesion and depressive symptoms. As shown in Model 1, migrants with a low perceived social cohesion had a higher CES-D score for urban-to-urban migrants [β = 1.17, Standard Error (SE) = 0.005, *p* < 0.001] and rural-to-urban migrants (β = 1.81, SE = 0.004, *p* < 0.001) compared to those with moderate-high social cohesion. As shown in Model 2, the addition of covariates reduced the β-coefficient for low social cohesion to 0.78 (SE = 0.005, *p* < 0.001) for urban-to-urban migrants and 1.74 (SE = 0.004, *p* < 0.001) for rural-to-urban migrants. Moreover, the addition of social adaptation to Model 3 further reduced the β-coefficient for low social cohesion to 0.66 (SE = 0.005, *p* < 0.001) for urban-to-urban migrants and 1.41 (SE = 0.004, *p* < 0.001) for rural-to-urban migrants, and the associations remained significant.

**Table 4:** Linear regression models for depressive symptoms (CES-D score; weighted).

		**Model 1**		**Model 2**		**Model 3**	
		β	**SE**	β	**SE**	β	**SE**
Urban-to-urban migrants (*N* = 1,227)	Low social cohesion	1.17^***^	0.005	0.78^***^	0.005	0.66^***^	0.005
	Fair or low social adaptation					1.55^***^	0.008
	Sobel test of mediation: *p* = 0.001						
	% Explained by the addition of social adaptation = 15.39%						
Rural-to-urban migrants (*N* = 1,357)	Low social cohesion	1.81^***^	0.004	1.74^***^	0.004	1.41^***^	0.004
	Fair or low social adaptation					1.94^***^	0.006
	Sobel test of mediation: *p* = 0.001						
	% Explained by the addition of social adaptation = 18.97%						

Source. 2017 Urbanization and New Migrant Survey.

Model 1 adjusted only for social cohesion. Model 2 added in adjustment of socio-demographic characteristics, health behaviors, health status, and migration characteristics, including gender, age, marriage status, education, income, smoking, alcohol consumption, physical activity, diabetes, hypertension, heart disease, reasons for migration, migration time, and city of residence. Model 3 added in the effects of social adaptation.

SE, Standard Error.

^*^p < 0.05, ^**^p < 0.01,

*** p < 0.001.

Formal mediation analysis was conducted to examine the mediating role of social adaptation on the associations between social cohesion and depressive symptoms in both groups. The Sobel mediation tests were significant for both groups at the *p* = 0.001 level. Social adaptation partially mediated the association between social cohesion and depressive symptoms by explaining 15.39% of its effect for urban-to-urban migrants and 18.97% of its effect for rural-to-urban migrants.

## Discussion

Using data from the “2017 Urbanization and New Migrant Survey,” we investigated the association between social cohesion and depressive symptoms among urban-to-urban and rural-to-urban migrants aged 18–65 in China and examined the mediating role of social adaptation. All three hypothesis were supported. The study findings demonstrated the significant mental health inequalities across urban-to-urban and rural-to-urban migrants. Also, we provided new knowledge to the field by identifying the protective role of social cohesion, as an important community-level factor, on depressive symptoms of migrants. Furthermore, this study identified the mediating role of social adaptation on the association between social cohesion and depressive symptoms. Our study has provided a scientific foundation for developing policy and research agenda to improve the mental health of migrants in China.

### Mental health care challenges: Disparities in depressive symptoms within migrants

Our findings that rural-to-urban migrants had a higher prevalence of depressive symptoms than urban-to-urban migrants confirmed the mental health inequalities within migrants. Whether they migrated from urban or rural may have implications for their housing registration status (*Hukou*), educational background, financial status, social capital, and sense of wellbeing. All these factors are related not only to the challenges they may face in the migration process but also to their abilities to overcome the challenges that could affect their probability of suffering from depressive symptoms.

The limited resources and opportunities due to their *Hukou* could partially explain rural-to-urban migrants' higher prevalence of depressive symptoms. Rural *Hukou* limits their access to both economic and social resources in the host city. In this situation, the social stress and social exclusion they experienced may have significant mental health implications (6, 10–12). Our results showed that a higher proportion of rural-to-urban migrants had less perceived social cohesion and fair or poor social adaptation than urban-to-urban migrants. Although China has gradually relaxed its policies on its *Hukou* system, it remains challenging for rural-to-urban migrants, especially those with low education, to settle permanently in the receiving city or are entitled to the same social benefits as urban residents (14, 35, 36). These inequalities could limit their opportunities for upward mobility and ultimately affect their mental health outcomes.

Our results also suggest that, among rural-to-urban migrants, involuntary migrated participants had higher depressive symptoms than those who voluntarily migrated. Several reasons may shed a slight on the findings. For example, these involuntary migrants may have limited social connections because family members are likely to be the only social network they can interact with. In addition, involuntary migrants have limited access to formal financial support, social capital, and health insurance, all are considered protective factors for maintaining good mental health (19).

### Social strategies: Building a higher cohesive community

Findings on the association between social cohesion and depressive symptoms are in line with previous studies which demonstrated that a higher level of neighborhood social cohesion is associated with better mental health outcomes (49, 50, 52, 55, 56).

Findings from linear regression models indicate that the level of depressive symptoms of rural-to-urban migrants is more dependent on social cohesion. It seems that social environmental context is even more important for rural-to-urban migrants' psychological wellbeing than for urban-to-urban migrants. In a host city, rural-to-urban migrants face much greater mental health risks than urban-to-urban migrants (6, 11, 17, 18); including (i) lack of urban *Hukou* that comes with a variety of economic and social resources (i.e., social security, unemployment insurance, and health insurance). (ii) Lack of sufficient level of education that could help them achieve a stable occupation and a higher socioeconomic position. (iii) Lack of sufficient social capital that is an important protective factor of psychological wellbeing. Although social capital appears to exist among rural-to-urban migrants, they have less-developed organizational social networks. Most of them rely on family members and friends from hometowns as primary personal networks (71). Thus, we speculate that they are more in need of support and help from a well-integrated community than urban-to-urban migrants. Tailored social strategies are needed to target rural-to-urban migrants for addressing mental health disparities among migrant population.

Additionally, our study identified the mediating role of social adaptation on the association between social cohesion and depressive symptoms for both groups. Reflecting the interactions between local residents and migrants, community-level social cohesion impacts whether migrants could “doing well” in the activities of daily living (59) and achieve good psychological wellbeing in the host city.

Our study findings demonstrate that social cohesion matters in mental health outcomes among migrants in China. Improving social cohesion could be an effective social strategy to promote the mental health of the migrant population. Social cohesion is undoubtedly influenced by governance, socio-economic and public policies (26). Thus, public programs and intervention strategies are needed to construct a more integrated community that serves both local residents and internal migrants.

Some limitations of this study need to be acknowledged. First, this study used cross-sectional survey data; we are only able to examine the associations of the variables of interest. First, this study used cross-sectional survey data; we are only able to examine the associations of the variables of interest. From a life-course perspective, the impact of migration and migration-related changes accumulate throughout the life-course; therefore, longitudinal studies are needed for future research. Second, this study was conducted in 10 cities, which is not representative of all migrant-receiving cities in China. Third, to better identify the pathways of social cohesion on depressive symptoms, we need to explore more mediating factors, such as social stress and social exclusion, in future studies. We thus call for future studies to develop and evaluate socio-behavioral interventions in addressing the mental health problems of the increasing number of internal migrants in China.

## Data availability statement

The raw data supporting the conclusions of this article will be made available by the authors. Requests to access these datasets should be directed to millie_qu@163.com.

## Ethics statement

The 2017 Urbanization and New Migrant Survey involving human participants were reviewed and approved by Ethics Committee of Shanghai University (ECSHU 2020-097). Written informed consent for participation was not required for this study in accordance with the national legislation and the institutional requirements.

## Author contributions

BW contributed to conceptualization, design, and manuscript preparation. XQu contributed to data analysis and manuscript preparation. XQi, JY, and HZ contributed to manuscript preparation. All authors have read and agreed to the published version of the manuscript.
